# Structural and functional characterization of human apolipoprotein E 72-166 peptides in both aqueous and lipid environments

**DOI:** 10.1186/1423-0127-18-4

**Published:** 2011-01-10

**Authors:** Yi-Hui Hsieh, Chi-Yuan Chou

**Affiliations:** 1 Department of Life Sciences and Institute of Genome Sciences, National Yang-Ming University, Taipei 112, Taiwan

## Abstract

**Backgrounds:**

There are three apolipoprotein E (apoE) isoforms involved in human lipid homeostasis. In the present study, truncated apoE2-, apoE3- and apoE4-(72-166) peptides that are tailored to lack domain interactions are expressed and elucidated the structural and functional consequences.

**Methods & Results:**

Circular dichroism analyses indicated that their secondary structure is still well organized. Analytical ultracentrifugation analyses demonstrated that apoE-(72-166) produces more complicated species in PBS. All three isoforms were significantly dissociated in the presence of dihexanoylphosphatidylcholine. Dimyristoylphosphatidylcholine turbidity clearance assay showed that apoE4-(72-166) maintains the highest lipid-binding capacity. Finally, only apoE4-(72-166) still maintained significant LDL receptor binding ability.

**Conclusions:**

Overall, apoE4-(72-166) peptides displayed a higher lipid-binding and comparable receptor-binding ability as to full-length apoE. These findings provide the explanation of diverged functionality of truncated apoE isoforms.

## Introduction

**Figure 1:**
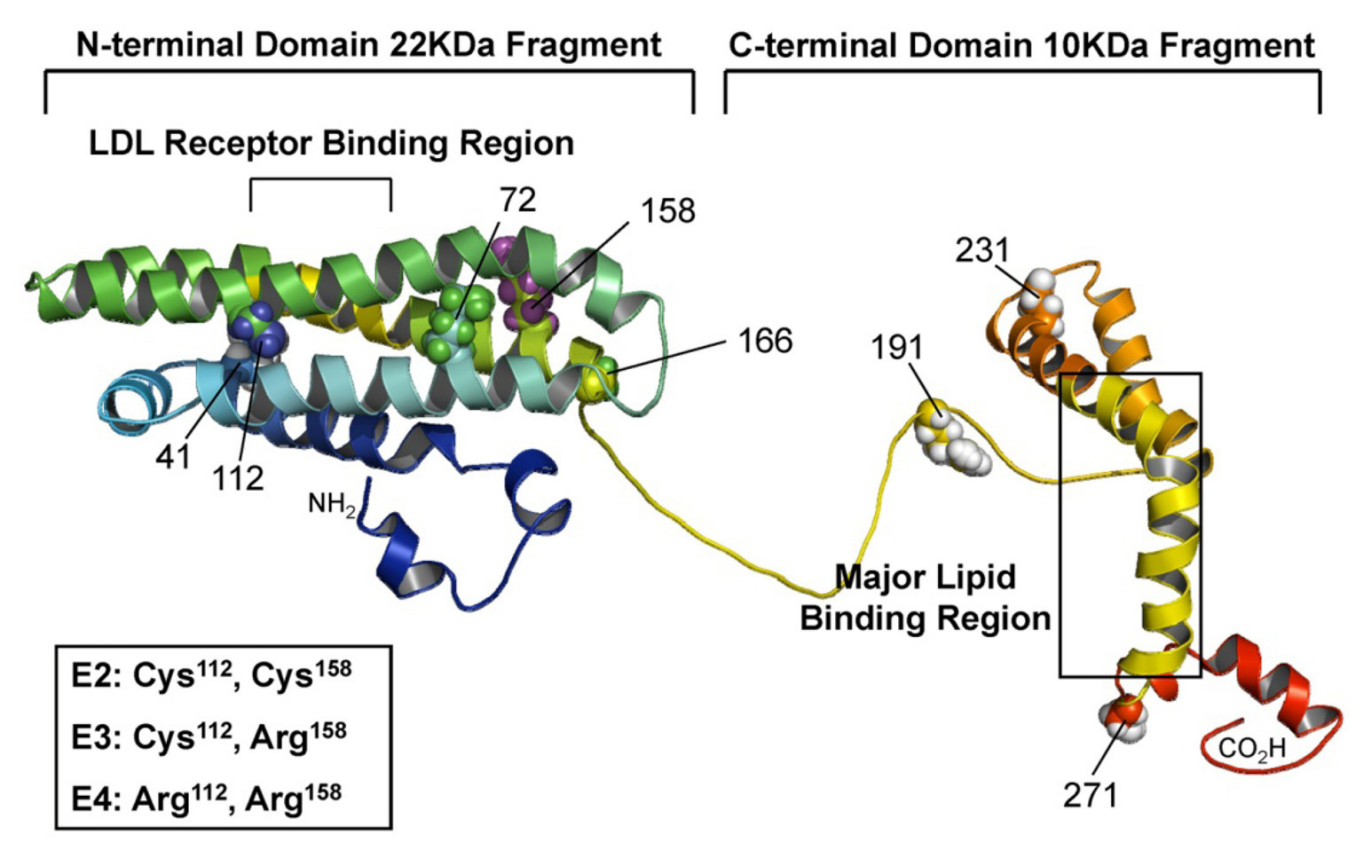
**Structure of human apoE proteins**. The model structure illustrating the full-length apoE with NT and CT domains. The structure was modified from apoE299_20K (S. Y. Sheu, unpublished data). The polymorphic sites (residues 112 and 158) that distinguished the three isoforms are highlighted. The picture was produced with PyMOL [46].

Human apolipoprotein E (apoE)^1 ^comprises 299 amino acids and there are three isoforms, apoE2, apoE3, and apoE4, encoded by the ε2, ε3, and ε4 genes, respectively. These isoforms differ from each other only at residues 112 and 158 i.e. Cys112 and Arg158 in apoE3, a cysteine at both positions in apoE2, and an arginine at both positions in apoE4 [1]. The amino-terminal (NT) domain of apoE contains four amphipathic α-helices and has pronounced kinks in the helices near the end of the four-helix bundle that correlates with the lipid binding ability (Figure 1) [2,3]. The residues between 140-150 in the fourth α-helix, comprising many basic amino acids, has been identified as the low-density lipoprotein receptor (LDLR) binding region [4], with the lipid binding region shown to be in the carboxyl-terminal (CT) domain [5,6]. The lipid association is required for high affinity binding of apoE to the LDLR because of the increased exposure of basic region on the fourth α-helix after interacting with lipids [7].

ApoE is involved in facilitating the transportation of plasma chylomicron remnant to the liver through either the remnant receptor or LDLR [8,9]. Owing to distinct domain interactions, apoE2 and apoE3 bind preferentially to small lipoproteins such as high-density lipoprotein (HDL), whereas apoE4 has a higher affinity to very-low-density lipoprotein (VLDL) [6,10]. Different to apoE3, apoE4 is prone to raise the plasma LDL to high levels and cause high oxidative stress that can facilitate atherosclerosis progression [11,12], whilst apoE2 is associated with type III hyperlipoproteinemia [13]. The ε4 allele is also associated with familial late-onset and sporadic Alzheimer's disease (AD) [14,15]. ApoE4 has been found to interact with beta-amyloid peptides (Aβ) and induce neurofibrillary tangle (NFT) formation [16,17]. It preferentially undergoes proteolysis to yield NT- and CT-truncated that interact with cytoskeletal components to form NFT-like inclusions in neuronal cells [16]. To understand the pathogenesis of different isofomic apoE, most studies are focused on the delineation of the structure and function characterization of the full-length apoE, varied length CT, or a "four α-helix bundle" NT domain [18-21].

In the present studies, we attempted to clarify the structural and functional consequences of NT- and CT-truncated apoE peptides, i.e. apoE-(72-166). This truncation still maintains the LDLR binding region, and removes the first two α-helices and the complete CT domain. The aim is to create a shorter but still functional apoE for potential therapeutic approach. Analytical ultracentrifugation was used to elucidate the quaternary structural properties of the three apoE-(72-166) isoforms. In the presence of lipid, the degree of apoE-(72-166) dissociation and extended conformation was significantly elevated. The functional assays conclude that apoE-(72-166) peptides still maintain comparable LDLR and higher lipid binding ability as to full-length apoE, particularly apoE4-(72-166). These findings suggest a crucial role of shorter NT-domain in the biological function of apoE and provide the basis for the explanation of diverged functionality of truncated apoE isoforms.

## Materials and methods

### Plasmids

The construction of pET-apoE2, apoE3, apoE4, apoE3-(72-166), and apoE4-(72-166) vectors were described previously [22]. The apoE2-(72-166) DNA fragment was amplified by PCR, and the forward primer was 5'-AAACATATGAAGGCCTACAAATCGGA, whereas the reverse primer was 5'-AACTCGAGGGCCCCGGCCT. The *Nde*I-*Xho*I digested apoE2-(72-166) cDNA was then ligated to the 5.2-kb *Nde*I-*Xho*I pET-29a(+) fragment.

### Expression and Purification of ApoE Proteins

Protein induction and purification procedures have been described previously [22,23]. Typical yields of the apoE-(72-166) proteins were 5-10 mg after purification from 1 liter of *E. coli *culture medium. The purity of all recombinant proteins was estimated by SDS-PAGE to be > 95% and the molecular mass of the apoE-(72-166) proteins was 12 kDa. The purified proteins were buffer-changed to phosphate buffered saline (PBS) (pH7.3) using Amicon Ultra-4 10-kDa centrifugal filter (Millipore).

### Preparation of Micelle Solution

Dihexanoylphosphatidylcholine (DHPC) has a critical micelle concentration of 16 mM, at which micelle monomers are formed containing 19 to 40 molecules based on ultracentrifugation, NMR, and small angle neutron scattering, respectively [24-26]. We used several concentrations of DHPC (5, 50, and 100 mM) to establish an appropriate lipid environment containing submicelles or micelles. In current studies, all experiments related to DHPC were executed at 20°C for the same lipid state.

### Circular Dichroism Spectroscopy

Circular dichroism (CD) spectra of the apoE-(72-166) peptides using a JASCO J-810 spectropolarimeter (Tokyo, Japan) showed measurements from 250 nm to 190 nm at 20°C in PBS (pH 7.3) with or without 50 mM DHPC. The protein concentration was 0.5 mg/ml. In wavelength scanning, the width of the cuvette was 0.1 mm. The far-UV CD spectrum data were analyzed with the CDSSTR program [27,28]. In this analysis, the α-helix, β-sheet, and random coil were split. To estimate the goodness-of-fit, the normalized root mean square deviation (NRMSD) was calculated.

### Unfolding of the ApoE-(72-166) Proteins in Guanidinium Chloride

ApoE-(72-166) proteins (0.1 mg/ml) with or without 50 mM DHPC were unfolded with different concentrations of GdnCl in PBS (pH 7.3) at 4°C overnight to reach equilibrium. The unfolding of the proteins was monitored by measuring the CD signal of 222 nm at 20°C and the width of the cuvette was 1 mm. The unfolding data were analyzed using thermodynamic models by global fitting of the measurements to the two-state unfolding model [29] as follows:

(1) $$y_{obs}=\frac{y_{N}+y_{U}•e^{-\left(\frac{\Delta G_{{\left(H_{2}O\right)}_{N\to U}}-m_{N\to U}\left[GdnCl\right]}{RT}\right)}}{1+e^{-\left(\frac{\Delta G_{{\left(H_{2}O\right)}_{N\to U}}-m_{N\to U}\left[GdnCl\right]}{RT}\right)}}$$

where *y_obs _*is the observed biophysical signal; *y_N _*and *y_U _*are the calculated signals of the native and unfolded states, respectively. *GdnCl *is the GdnCl concentration, and $\Delta G_{(H_{2}O)N\to U}$ is the free energy change for the *N→U *process. *m_N→U _*is the sensitivity of the unfolding process to a denaturant concentration.

### Sedimentation Velocity

Sedimentation velocity (SV) experiments were performed with an XL-A analytical ultracentrifuge (Beckman, Fullerton, CA) as described previously [23]. All studies were performed at 20°C with a rotor speed of 42,000 rpm in PBS (pH 7.3) with or without DHPC. The protein concentration was 0.5 mg/ml. Multiple scans at different time periods were then fitted to a continuous c(s) distribution model using the SEDFIT program as described previously [30,31]. All continuous size distributions were calculated using a confidence level of *p *= 0.95, a best fitted average anhydrous friction ratio (f_r_), a resolution value N of 200, and sedimentation coefficients between 0 and 20 S. For the data fitting of apoE-(72-166) in PBS and 5 mM DHPC, the partial specific volume was set to 0.73 for proteins species. Differently, for those in 50 and 100 mM DHPC, the value was set to 0.86 because the influence of DHPC micelle. Previous studies have suggested that DHPC's partial specific volume is 0.99 ml/g [32]. According to our calculation, higher partial specific volume will lower the best fitted average f_r_, while the c(s) distribution will not have any difference.

### Sedimentation Equilibrium

Sedimentation equilibrium (SE) experiments were performed with six-channel *epon *charcoal-filled centerpieces as described previously [22]. The cells were then mounted into an An-60 Ti rotor and centrifuged at 10,000 rpm, 15,000 rpm, and 20,000 rpm, respectively, each for 18 h at 20°C. Ten A_280 nm _measurements with a time interval of 8-10 min were performed for each different rotor speed to check the equilibrium state. The SV and SE spectrum of each apoE-(72-166) protein under the same environments were combined and then fitted to a global discrete species model using SEDPHAT program as described previously [22,33].

### DMPC Turbidity Clearance Assay

The preparation of DMPC (Sigma, St Louis, MO) multilamellar vesicles (mLV) has been described previously [22,34-36]. ApoE (250 μg) was added to DMPC mLV solution (0.5 mg/ml) in a quartz cuvette which had been preincubated at 24°C in a Perkin-Elmer Lambda 35 spectrophotometer with water circulated temperature control. Vesicle solubilization was monitored as a decrease in the absorbance at 325 nm. The time course of the clearance measurements were fitted by nonlinear regression to the biexponential decay equation,

(2) $$Y=A\cdot e^{-k_{1}\cdot t}+B\cdot e^{-k_{2}\cdot t}+C$$

where Y is the absorbance at 325 nm and *k*, *k_1 _*or *k_2 _*are the rate constants for different kinetic phases of the solution clearance. *A *and *B *are the changes in turbidity for different phases (pool sizes), *t *is the time, and *C *is the remaining turbidity at the completion of the reaction.

### In vitro VLDL Binding Assay

ApoE proteins were incubated with apoE(-) mice serum at 37°C. The molar ratio of apoE and VLDL was 1:1 for the apoE and 5:1 for the apoE-(72-166) proteins. After a 4 h incubation, the apoE-VLDL particles and free apoE were separated by NaBr density ultracentrifugation (Optima L-90K ultracentrifuge, Beckman). At first, the density of serum was corrected to 1.211 g/ml by adding NaBr. The serum solution was then loaded into 10-ml ultracentrifuge bottles (polycarbonate, Beckman, Fullerton, CA) and centrifugation was performed for 24 h with a rotor (Beckman 70.1 Ti) speed of 44,000 rpm at 4°C. After centrifugation, the lipoproteins (HDL, LDL, and VLDL) float on the solution surface and can be recovered by pipetting. The binding of apoE-VLDL was then confirmed by lipoprotein electrophoresis (hydragel lipo + Lp(a) K20, Sebia) at 50 V, a current of 25 mA, and a power setting of 5 W for 3 h. The LDL, VLDL, and HDL molecules were separated by their charge and the VLDL band was shifted with the binding of apoE proteins.

### LDLR Binding Assay

The detailed procedures for the LDLR binding assay have been described previously [22,37,38]. Briefly, human hepatoblastoma cells (HepG2) were incubated in DMEM with 10% fetal bovine serum at 37°C followed by incubation with DMEM containing ^3^H-LDL and different receptor binding competitors (apoE proteins) at 4°C for 2 h. After washing, cells were released, lysed, and the radioactivity was determined using a liquid scintillation counter (Beckman, Fullerton, CA).

## Results and Discussions

### Secondary Structures of the apoE-(72-166) peptides is well organized and α-helical dominant

Based on the far-UV CD measurements we made, apoE2-, apoE3-, and apoE4-(72-166) peptides maintained 49, 48, and 53% α-helical structure in PBS; and 47, 49, and 45% in DHPC micellar solution, respectively (Additional file 1: Figure S1A, B, and Table S1). The structure of apoE-(72-166) peptides was estimated to be α-helix dominant in both aqueous and DHPC micellar solution, although the content of α-helix was lower than the value from the solved crystal structure of NT domain (residues 23-166, pdb code: 1LPE), which is 74% [39]. The shorter length of our peptides and lower protein concentration used in CD may be the reason. Overall, the content of α-helix in all three isoforms did not change too much in the two environments, while the content of β-strand increased by 8-10% in DHPC micellar solution. Consequently, their random coil decreased by 1-11%. These data indicated that in the aqueous or DHPC micellar solution, the secondary structure of apoE-(72-166) was well organized and did not show very significant isoformic difference.

### The secondary structure of apoE-(72-166) was more stable in the solution containing DHPC micelles

To delineate the structural stability of the apoE-(72-166) peptides with or without DHPC, the GdnCl denaturation experiments were executed. The denaturation of the three apoE-(72-166) proteins followed a two-state transition (Additional file 1: Figure S1C, D). Our experimental data was then fitted using equation 1 to calculate the change of free energy, m value, and [GdnCl]_0.5 _(Table 1). In the presence of DHPC micelle, the m value of the three isoforms showed a significant decrease, while $\Delta G_{(H_{2}O)N\to U}$ did not. It resulted in the [GdnCl]_0.5 _of the three isoform increased by 0.8-0.86 M, respectively, comparing to those in PBS. These differences suggested that the secondary structure of apoE-(72-166) was more stable in the solution containing DHPC micelles. Recent studies for apolipoprotein C-II amyloid fibrils have shown similar phenomenon that phospholipid interactions can stabilize regular secondary structure formations and molecular-level polymorphisms [40].

**Table 1:** Guanidine hydrochloride denaturation of apoE-(72-166) proteins with and without DHPC

Buffer	Protein	$\Delta G_{(H_{2}O)N\to U}$^a ^(kcal mol^-1^)	m (kcal mol^-1 ^M^-1^)	[GdnCl]_0.5_(M)
PBS	apoE2-(72-166)	1.93 ± 0.14	1.37 ± 0.09	1.40 ± 0.14
	apoE3-(72-166)	1.71 ± 0.18	1.51 ± 0.13	1.13 ± 0.15
	apoE4-(72-166)	1.52 ± 0.20	2.45 ± 0.27	0.62 ± 0.11

PBS + 50 mM DHPC	apoE2-(72-166)	1.89 ± 0.24	0.84 ± 0.11	2.25 ± 0.41
	apoE3-(72-166)	2.18 ± 0.23	1.13 ± 0.11	1.93 ± 0.28
	apoE4-(72-166)	1.30 ± 0.26	0.88 ± 0.15	1.48 ± 0.39

^a^The denaturation data were analyzed by the two-state unfolding model (eq. 1). The R_sqr _of each result was from 0.975 to 0.997.

Similar to full-length apoE proteins in a lipid-free solution [20], the differences between the apoE-72-166 protein isoforms in terms of structural stability was in the order of apoE2 > apoE3 > apoE4. Previous structural studies indicated that Cys112 of apoE3 is partially buried between helices 2 and 3, while Arg112 of apoE4 could be easily accommodated by filling the solvent region surrounding the helix pair [39]. This variation may cause apoE4 more unstable. By the way, it further suggests that the structure of apoE4-(72-166) is more easily opened and exposed more hydrophobic residues. Indeed, by 1-anilino-8-naphthalenesulfonic acid titration analysis (our unpublished data), the apoE4-(72-166) shows the highest hydrophobic exposure, which can further explain the highest ability of DMPC turbidity clearance of apoE4-(72-166) (see below). Differently but not surprisingly, apoE-(72-166) displayed a two-state transition, whereas full-length apoE showed a three-state unfolding process. We also found that the [GdnCl]_0.5 _values for apoE2-, and apoE3-(72-166) were about 1.1-1.4 M, very close to the [GdnCl]_0.5,N-I _of full-length apoE2 and apoE3. However, the [GdnCl]_0.5 _of apoE4-(72-166) was only 0.6 M, which was lower than the [GdnCl]_0.5,N-I _measurement of full-length apoE4 (0.9 M). Remarkably, the relatively unstable apoE4-(72-166) fragment still possessed a 53 % α-helical structure. More detailed structural analysis may be required to explain the reciprocal low structural stability and high α-helical content of apoE4-(72-166) in aqueous environment.

### Our SV experiments and c(s) distribution analysis demonstrate a different species distribution of apoE-(72-166) in aqueous and lipid environments

**Figure 2:**
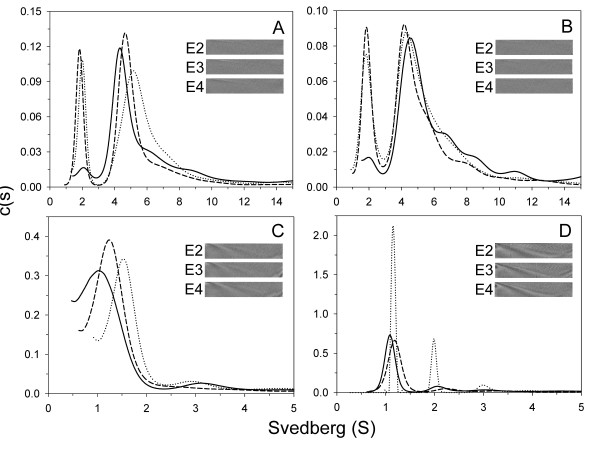
**c(s) distribution of apoE-(72-166) proteins in PBS with or without DHPC**. The sedimentation velocity data was fitted with the SEDFIT program using the continuous c(s) distribution model [30]. The fitted curves for apoE2-, apoE3-, and apoE4-(72-166) are shown as dotted, dash, and solid lines, respectively. Panels A-D: proteins were in PBS, and with 5 mM, 50 mM, or 100 mM DHPC, respectively. Insets, grayscale of the residual bit map showing the quality of data fitting.

In PBS, apoE-(72-166) proteins showed a distribution pattern of two major species (Figure 2A). The first of these showed a sedimentation coefficient distribution of 20 % for apoE2-(72-166) and 23 % for apoE3-(72-166) at s = 2.0, but only 6 % for the same species of apoE4-(72-166). The second major species was a broad peak at s = 3.5 to 6.5, with a total occupancy of 46 % for apoE2-(72-166), 55 % for apoE3-(72-166), and 59 % for apoE4-(72-166). This region may be the result of a contribution by multi-oligomers. Besides, there were 22-35 % distribution belonged to large aggregated forms. In the 5 mM DHPC submicellar solution, the small species (s = 2) of the three apoE-(72-166) increased by 1.3 to 4 % (Figure 2B), whereas the major species at s = 3.5-6.5 decreased by 2 to 8 %. It suggested that submicellar DHPC can induce the dissociation of apoE-(72-166) peptides but not very significantly. In 50 mM DHPC, 76 to 82 % of the apoE-(72-166) proteins dissociated to a species at s = 1.2-1.5 (Figure 2C). Finally, whilst apoE2-(72-166) maintained a two species distribution (s = 1.1 and 2.0) in 100 mM DHPC, its apoE3 and apoE4 counterparts maintained a single major species at s = 1.1 (Figure 2D). Furthermore, by c(s) distribution analysis we found that the average f_r _of apoE-(72-166) in PBS was around 1.3-1.5, but in 5-50 mM DHPC was around 1.7-1.8, which increased to 1.7-2.1 in 100 mM DHPC (partial specific volume at 0.86). These differences indicated that when the DHPC concentration increases, apoE-(72-166) not only displays a dissociation tendency, but also adopts a more elongated conformation.

### The mass variation of the apoE-(72-166) in PBS and in DHPC was analyzed by global discrete species model

**Figure 3:**
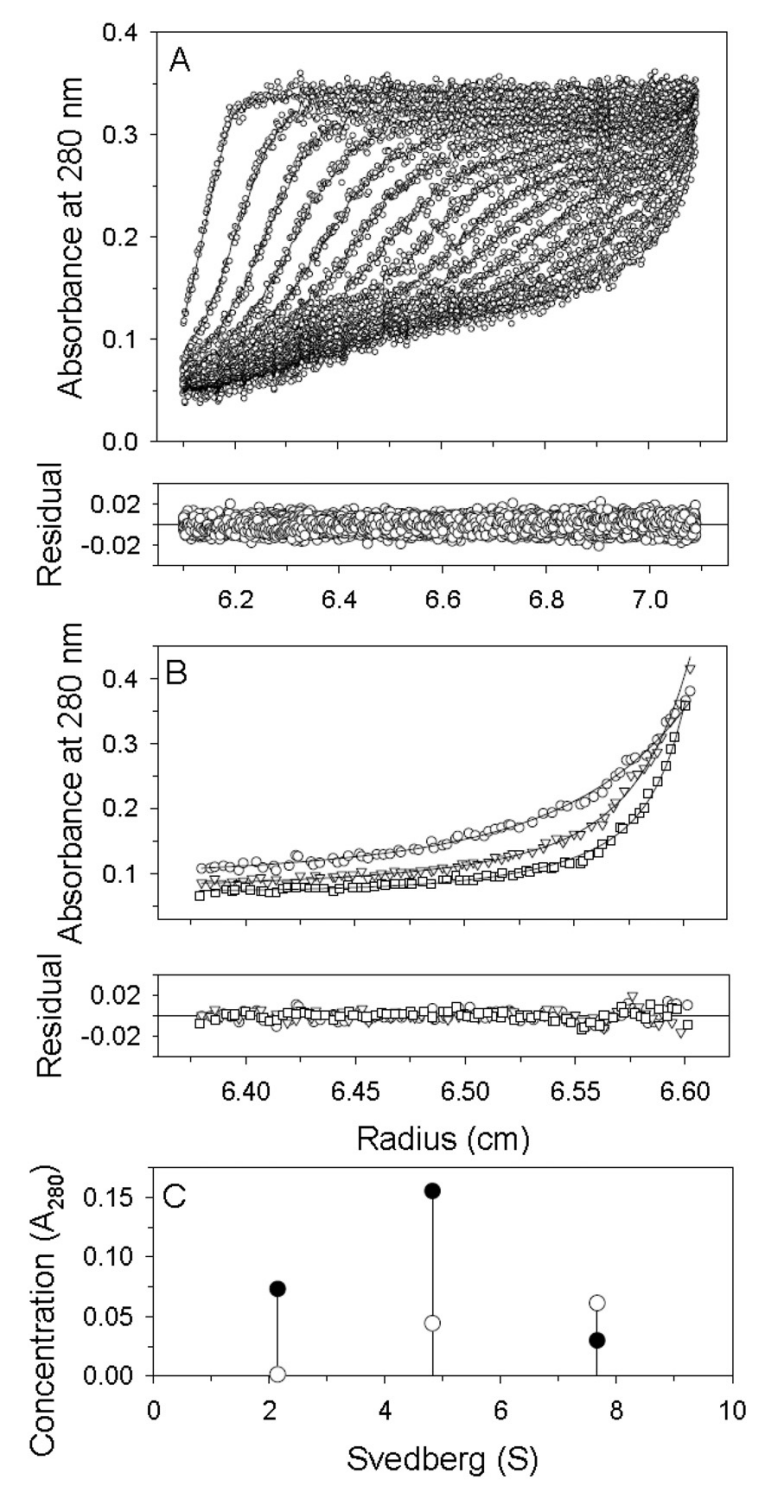
**Global analysis of the apoE3-(72-166) proteins in PBS (pH 7.3)**. The SV experiment (A) was centrifuged to 42,000 rpm (circles) at 20°C for 4 h. The speed of centrofugation for SE experiments (B) was 10,000 rpm (circles), 15,000 rpm (triangles), and 20,000 rpm (squares) at 20°C each for 18 h. The solid lines in A-B are the best fit distributions from global analysis of the three discrete species model by SEDPHAT according to eq. 4. The molar mass and sedimentation coefficients of the species were floated and fitted. The residuals of each fit are shown below the panels and have a local RMSD for each channel of 0.0054 (A) and 0.0050 (B). The discrete species distribution of apoE3-(72-166) from SV (closed circles) and SE (open circles) are shown in C. The parameters by best fit are shown in Table 3.

To further clarify the mass variation of the three apoE-(72-166) peptides in PBS and also in the presence of DHPC, SE experiments were performed. The SE and SV data were combined and globally fitted to a multiple discrete species model using SEDPHAT. Figure 3 showed the best-fit results of apoE3-(72-166) in PBS. According to the results of c(s) distribution (Figure 2), the data were adequately described and fitted by a three (those in PBS and 5 mM DHPC) and two (those in DHPC micelle) discrete species model, respectively. The best-fit results are summarized in Table 2. The calculated local concentration and sedimentation coefficient of each discrete species showed a similar content to those in c(s). Most major species detected in SV were also detected in SE experiments. In the aqueous PBS solution, apoE-(72-166) peptides showed a major species of dimer, tetramer (for apoE4-(72-166)) or hexamer (for apoE2- and apoE3-(72-166)), and large aggregates, respectively, which indicated a significant polymerization. In the 5 mM DHPC submicellar solution, the content of each species did not show significant change, although the hexamer of apoE2- and apoE3-(72-166) dissociated to tetramer. It may suggest that apoE-(72-166) peptides begin to dissociate, which is consistent with the observation by c(s). In the presence of 50 mM DHPC micelles, all three apoE-(72-166) proteins maintained a major species of 19-20 kDa, which may be a complex structure of a monomeric apoE-(72-166) peptides (12 kDa) with a smaller DHPC micelle (20 molecules, 9 kDa). As a ellipsoid micelle with 20 DHPC molecules, the radius of gyration of the fatty acyl core region is 15.6 Å [41], whose circumference is about 100 Å, just identical to the length of apoE-(72-166) α-helical region. Besides, by SE experiments, apoE-(72-166) showed a major species of dimer (for apoE3- and apoE4-(72-166)) or tetramer (for apoE2-(72-166)) with a larger DHPC micelle (40 molecules, 18 kDa). As a micelle with 40 DHPC molecules, which has surface area of 2 times, the circumference will be about 140 Å. It may result in that apoE-(72-166) peptides do not form a complete belt around the micelle but are staggered at a suitable angle to each other [42]. Similarly, most apoE-(72-166) proteins in the presence of 100 mM DHPC micelles were found to have a major complex species of monomeric peptides with a micelle. The peptide-lipid complex with higher molar mass was also found by SE experiments.

**Table 2:** Global discrete species analysis of apoE-(72-166) with different environments^a^

**In**^**b**^	ApoE2-(72-166)			ApoE3-(72-166)			ApoE4-(72-166)		
	
	**S**^**c **^**(Svedberg)**	**M**^**d **^**(kDa)**	**Local C of SV and SE (A**_**280**_**)**^**e**^	**S**^**c **^**(Svedberg)**	**M**^**d **^**(kDa)**	**Local C of SV and SE (A**_**280**_**)**^**e**^	**S**^**c **^**(Svedberg)**	**M**^**d **^**(kDa)**	**Local C of SV and SE (A**_**280**_**)**^**e**^
PBS	2.2	19	0.07, 0.10	2.1	18	0.07, 0	2.7	24	0.02, 0
	5.3	67	0.17, 0	4.8	68	0.16, 0.04	4.7	51	0.18, 0.05
	7.8	186	0.05, 0.11	7.7	251	0.03, 0.06	8.6	210	0.05, 0.12
5 mM DHPC	2.3	25	0.07, 0.08	2.5	21	0.08, 0.08	2.2	22	0.01, 0
	4.9	49	0.18, 0	4.9	52	0.15, 0	4.8	51	0.17, 0.07
	8.2	190	0.04, 0.09	8.4	232	0.03, 0.06	8.5	217	0.04, 0.19
50 mM DHPC	1.3	19	0.35, 0.13	1.2	20	0.34, 0	1.1	19	0.29, 0
	4.3	71	0.01, 0.15	3.1	40	0.02, 0.15	3.8	45	0.03, 0.14
100 mM DHPC	1.2	30	0.31, 0	1.0	19	0.32, 0.08	0.8	20	0.27, 0.04
	3.3	49	0.05, 0.17	2.3	50	.0, 0.10	3.0	58	0.01, 0.11

^a ^The SV and SE experiments of apoE-(72-166) were combined and fitted to the global discrete model by SEDPHAT [33]. The best-fit local root mean square errors of SE were from 0.0041 to 0.0118 and those of SV were from 0.0054 to 0.0088.

^b ^The partial specific volume was set by 0.73 in PBS and 5 mM DHPC and by 0.86 in 50 and 100 mM DHPC (see materials and methods for detail).

^c, d, e ^Best-fit calculated sedimentation coefficients (s), molar mass (M), and local concentrations (C) of different species were shown. The local concentration of SV was from the discrete distribution of SV and that of SE was from the SE experiments. The concentration units were signal units (A_280_).

Nevertheless, our study demonstrates that DHPC may provide a lipid or hydrophobic rich environment that will facilitate the maintenance of a dissociated and extended conformation for apoE-(72-166). This tendency also positively correlates with the increasing concentration of DHPC.

### Protein-lipid interactions and Protein-LDLR binding of ApoE-(72-166) Proteins

**Figure 4:**
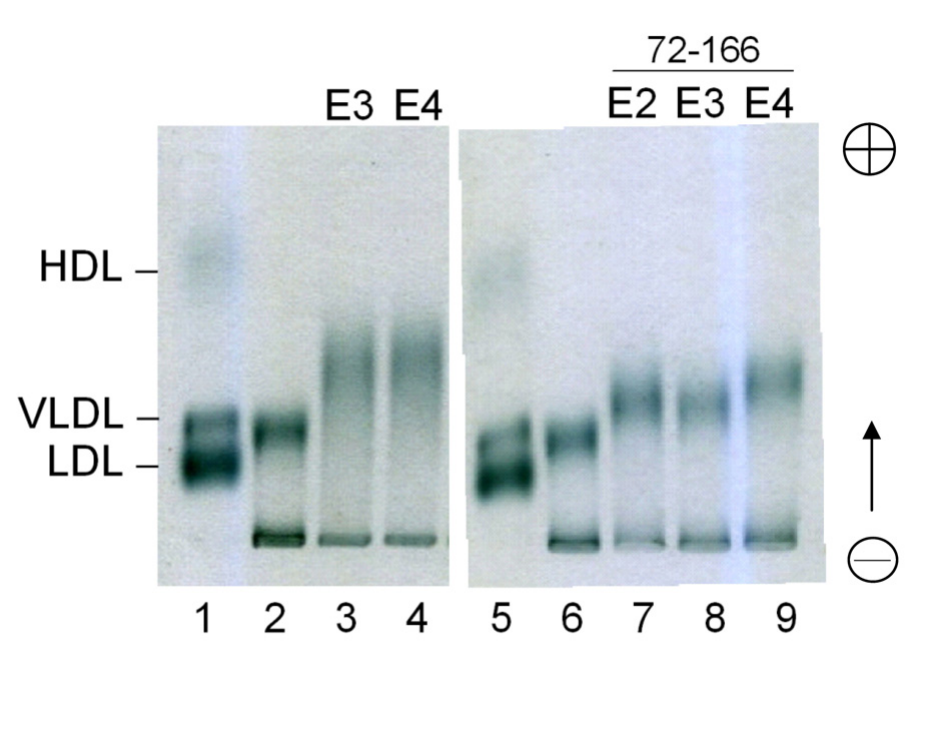
**Lipoprotein electrophoresis of apoE-VLDL particles**. Various apoE proteins were incubated with apoE(-) mice serum at 37°C for 4 h, respectively. After removing the free proteins by NaBr density ultracentrifugation, the VLDL particles were checked by zone electrophoresis (separation by charge). Lanes 1 and 5, human serum sample; lane 2 and 6, apoE(-) mice serum sample; lane 3-4 and 7-9, apoE(-) mice serum incubated with full length apoE3 and apoE4, and with apoE2-, apoE3-, and apoE4-(72-166) proteins, respectively. The VLDL bands were shifted with the binding of apoE proteins. Detailed procedures are described in Materials and Methods.

To identify and compare the lipid binding ability of the three apoE-(72-166) peptides, we assessed the DMPC turbidity clearance ability of apoE2-(72-166) (Additional file 1: Figure S2). Compared with the other two isoforms [22], apoE4-(72-166) had the highest DMPC turbidity clearance ability. By fitting to biexponential decay model (Eq. 3), it suggested that the rate constants of apoE4-(72-166) in both phase were 4-13 times faster than apoE2 and apoE3 counterparts and 99.9% turbidity was removed, which indicated that all DMPC mLV have been solubilized by apoE4-(72-166) (Additional file 1: Table S2). Furthermore, to evaluate if apoE-(72-166) peptides can bind to lipoprotein particles, the *in vitro *binding experiment of apoE(-) mice VLDL with the apoE proteins was analyzed using zone electrophoresis, which can separate the lipoproteins by their charge [43]. In these experiments, the interaction of VLDL and apoE proteins increased the charge of VLDL particles, resulting in the migration of VLDL band (lane 2 vs. lane 3-4 in Figure 4). Remarkably, the three apoE-(72-166) proteins also showed significant VLDL shifts (lane 6 vs. 7-9 in Figure 4), which indicated that the region containing residues 72-166 was sufficient for binding VLDL.

In our previous study, we have evaluated the LDLR binding ability of apoE3-(72-166) and apoE4-(72-166) [22]. Here we further analyzed the LDLR binding ability of apoE2-(72-166) peptides as a comparison with apoE3 and apoE4 counterparts (Additional file 1: Figure S3). As previously, we employed HepG2 cells as the LDLR carriers [22]. ^3^H-LDL was used as the ligand and the apoE proteins with or without DMPC were therefore the competitors. Overall, apoE-DMPC complex showed better ^3^H-LDL competition than apoE. Among the three isoforms, apoE4-(72-166)-DMPC complex decreased the ^3^H-LDL binding by 55%, comparing with 19% for apoE2-(72-166)-DMPC and 26% for apoE3-(72-166)-DMPC. At the same dose, apoE4-(72-166)-DMPC maintained almost identical LDLR binding ability to that of full length apoE-DMPC, while those of apoE2- and apoE3-(72-166) were significantly lower [22]. This indicated that alone of the three isoforms, only apoE4-(72-166) did not lose its LDLR binding ability. Comparing to the apoE2 and apoE3 counterpart, apoE4-(72-166) shows the highest lipid binding ability (Additional file 1: Figure S2 and Table S2). The lipid association is required for high affinity binding of apoE to the LDLR because of the increased exposure of basic region on the fourth α-helix after interacting with lipids [7].

## Conclusion

**Figure 5:**
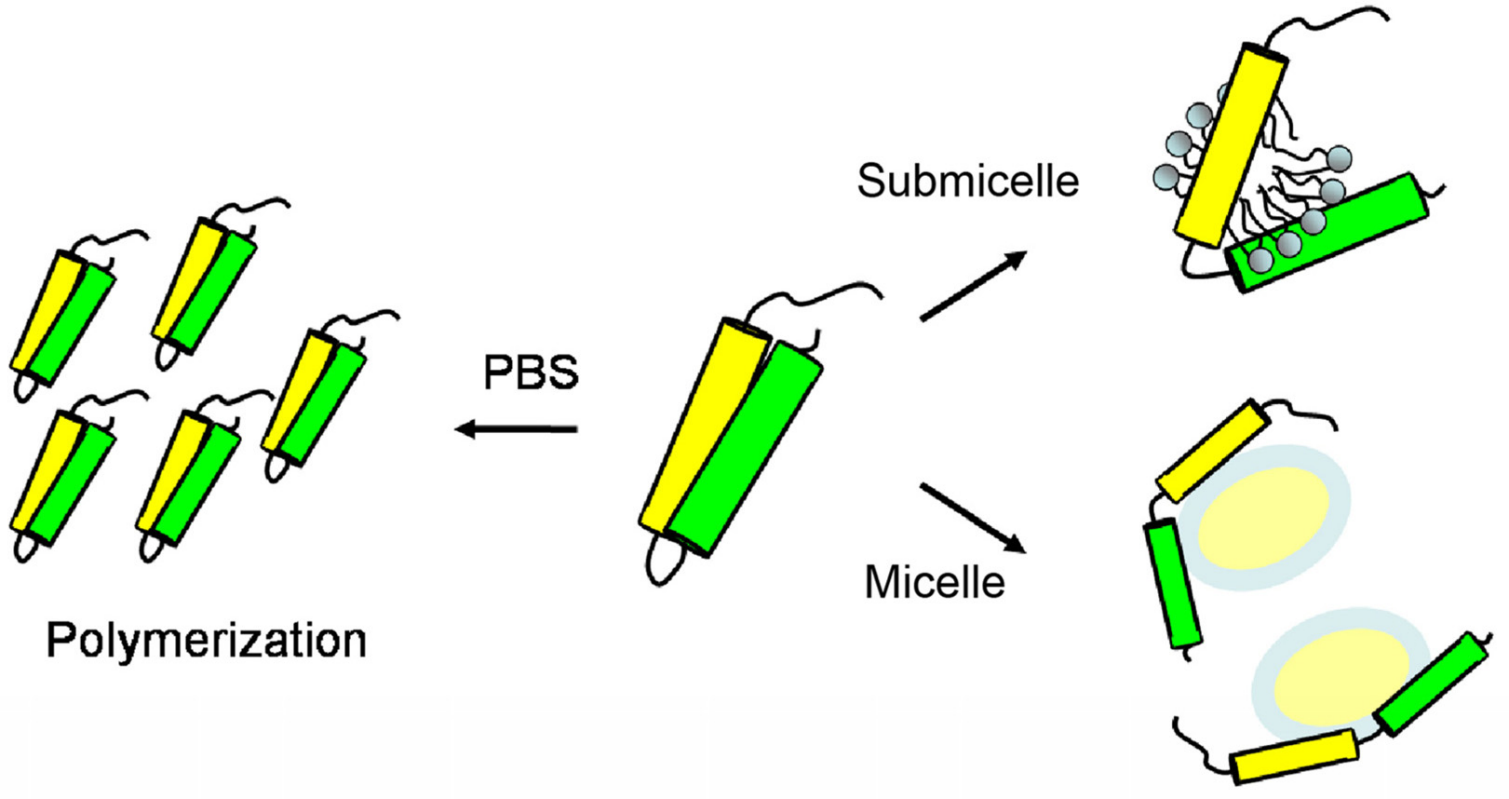
**Proposed model**. Schematic diagram for the apoE-(72-166) peptides in PBS, 5 mM DHPC submicelles, and 50 mM DHPC micelles. The yellow and green cylinders show the positions of residues 87-124 and 131-162, respectively.

To illustrate the interaction of apoE-(72-166) peptides with lipids, a model for apoE-(72-166) in PBS with or without DHPC is proposed (Figure 5). ApoE-(72-166) was found to be prone to polymerize in PBS. When apoE-(72-166) interacts with DHPC submicelles, these DHPC molecules will intercalate into its hydrophobic region causing hydrophobic exposure. In the DHPC micellar solution, apoE-(72-166) will dissociate and interact to a DHPC micelle with an extended conformation. We demonstrate herein that unlike the four α-helical bundle NT domain which maintains a stable monomer [22], apoE-(72-166), as a less structured peptide, may have less lateral contacts and tend to aggregate in PBS, but dissociates at the existence of DHPC micelle which may stabilize back these contacts. Besides, the truncated apoE peptides, especially apoE4-(72-166), still displays the comparable LDLR binding and higher lipid binding abilities as to full-length apoE [22]. Compared with a fused peptide which may have shorter half-life [44,45], the remarkable lipid binding and LDLR binding avidity of the apoE4-(72-166) suggests the possible feasibility for designing a competitive peptide against atherosclerosis or AD.

## Abbreviations

^1^Aβ: β-amyloid peptide; AD: Alzheimer's disease; apoE: apolipoprotein E; CD: circular dichroism; CT: carboxyl-terminal; DHPC: dihexanoylphosphatidylcholine; DMPC: dimyristoylphosphatidylcholine; f_r_: frictional ratio; GdnCl: guanidinium chloride; HDL: high-density lipoprotein; LDLR: low-density lipoprotein receptor; Meq: equivalent molar mass; mLV: multilamellar vesicles; NFT: neurofibrillary tangle; NRMSD: normalized root mean square deviation; NT: amino-terminal; PBS: phosphate buffered saline; SE: sedimentation equilibrium; SV: sedimentation velocity; VLDL: very-low-density lipoprotein

## Competing interests

The authors declare that they have no competing interests.

## Authors' contributions

YHH carried out most experiments and helped to draft the manuscript. CYC conceived the study, participated in experimental design, analyzed the AUC data, and drafted and revised the manuscript. Both authors read and approved the final manuscript.

## Supplementary Material

Additional file 1 **Tables S1 and S2. Figures S1-S3**. Click here for file
