# Supplementary material for: Structural and functional characterization of human apolipoprotein E 72-166 peptides in both aqueous and lipid environments
Source: J Biomed Sci. 2011 Jan 10;18(1):4. doi: 10.1186/1423-0127-18-4 (PMC3022805; doi:10.1186/1423-0127-18-4)
Supplement: Additional file 1 — Tables S1 and S2. Figures S1-S3. [file 1423-0127-18-4-S1.DOC]

**Additional Files**

**Structural and functional characterization of human apolipoprotein E 72-166 peptides in both aqueous and lipid environments**

Yi-Hui Hsieh and Chi-Yuan Chou^1^

Department of Life Sciences and Institute of Genome Sciences, National Yang-Ming University, Taipei 112, Taiwan

^1^Corresponding author: Dr. Chi-Yuan Chou, Department of Life Sciences and Institute of Genome Science, National Yang-Ming University, 155 Li-Nong St., Sec. 2, Taipei 112, Taiwan, R.O.C.

Tel.: +886-2-2826-7168, Fax: +886-2-2820-2449

E-mail: [cychou@ym.edu.tw](mailto:cychou@ym.edu.tw)

Table S1: Secondary structure of apoE-(72-166) proteins with and without DHPC

| Buffer | Protein | Secondary structure (%) ^a^ | | | NRMSD value |
| --- | --- | --- | --- | --- | --- |
|  |  | α-Helix | β-Sheet | Random coil |  |
| PBS | apoE2-(72-166) | 49 ± 2 | 10 ± 4 | 40 ± 6 | 0.035 |
|  | apoE3-(72-166) | 48 ± 2 | 11 ± 3 | 40 ± 5 | 0.028 |
|  | apoE4-(72-166) | 53 ± 3 | 15 ± 3 | 32 ± 5 | 0.011 |
| PBS + DHPC^b^ | apoE2-(72-166) | 47 ± 2 | 19 ± 2 | 35 ± 4 | 0.007 |
|  | apoE3-(72-166) | 49 ± 2 | 21 ± 2 | 29 ± 4 | 0.005 |
|  | apoE4-(72-166) | 45 ± 3 | 24 ± 3 | 31 ± 4 | 0.004 |

^a^ Percentage of the secondary structure conformations was calculated using the CDSSTR [28]. The experiments were repeated at least twice with moderate standard error.

^b^ 50 mM DHPC was used.

Table S2: Kinetic parameters for the 16-h clearance time courses of DMPC mLV by

| Biexponential Decay Model^b^ | | | | | | | | |  |
| --- | --- | --- | --- | --- | --- | --- | --- | --- | --- |
|  | | Rapid Phase | | |  | Slow Phase | | | |
| Protein^c^ | Remaining turbidity^d^ | Rate  constant^e^  (10^-2^min^-1^) | Pool size^e^  (fraction) | Flux^f^ (10^-2^ fraction/min) |  | Rate  constant^e^  (10^-2^min^-1^) | Pool size^e^  (fraction) | Flux^f^ (10^-2^ fraction/min) | |
| ApoE2_72-166_ | 0.163 ± 0.002 | 1.36 ± 0.02 | 0.234 ± 0.002 | 0.32 |  | 0.20 ± 0 | 0.611 ± 0.001 | 0.12 | |
| ApoE3_72-166_ | 0.069 ± 0.003 | 2.66 ± 0.02 | 0.592 ± 0.002 | 1.57 |  | 0.17 ± 0 | 0.322 ± 0.002 | 0.05 | |
| ApoE4_72-166_ | 0.001 ± 0 | 9.98 ± 0.13 | 0.624 ± 0.006 | 6.23 |  | 2.25 ± 0.02 | 0.408 ± 0.006 | 0.92 | |

apoE-(72-166) peptides^a^

^a^ Data of apoE3-(72-166) and apoE4-(72-166) are from [22].

^b^ The results were best fitted to the biexponential decay equation (Eq. 4).

^c^ The combination ratio of DMPC mLV and different apoE proteins was 2:1 (w/w).

^d^ The fitted remaining turbidity at completion of the reaction.

^e^ Mean ± standard error.

^f^ Value of product (rate constant x pool size) for each time course.


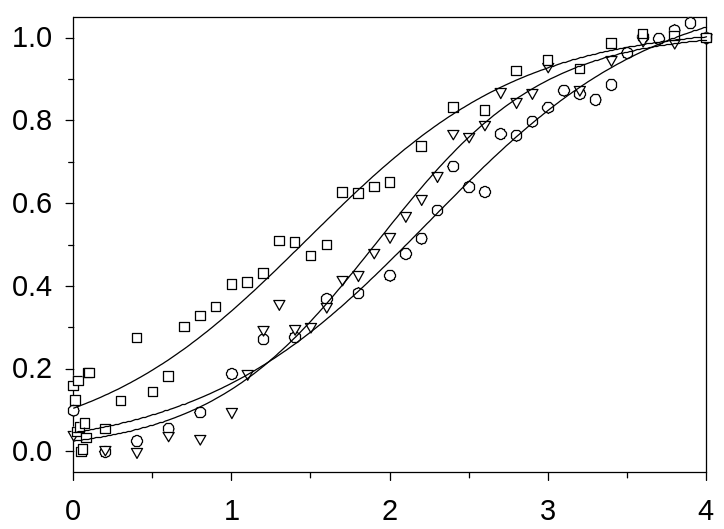

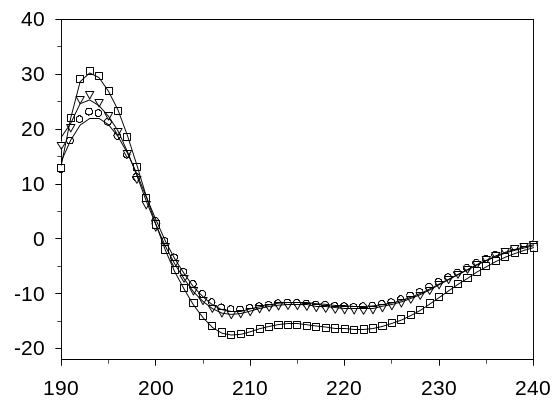

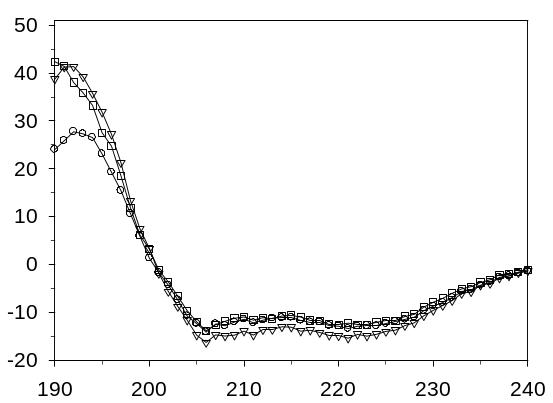


[GdnCl] (M)

MRE (x10^3^ dge cm^2^ dmol^-1^)

A

B

D

Wavelength (nm)


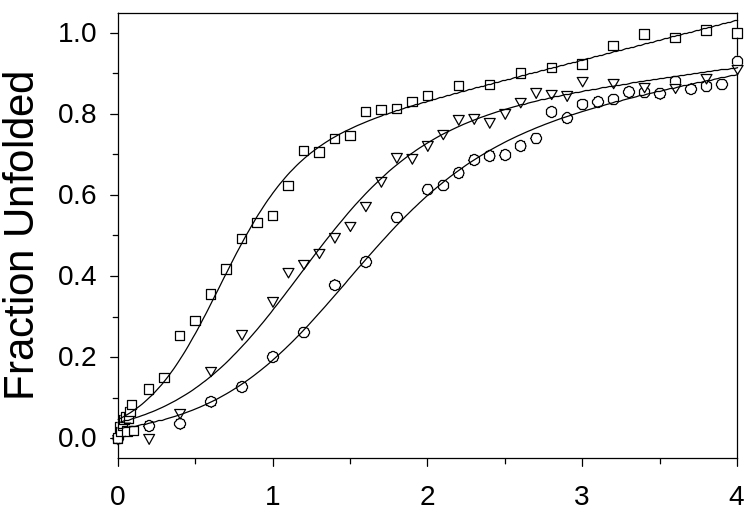


C

**Figure S1.** **CD spectra (A and B) and GdnCl denaturation analysis (C and D) of apoE-(72-166) proteins with or without DHPC.** The proteins (0.5 mg/ml for wavelength scan and 0.1 mg/ml for GdnCl denaturation) were suspended in PBS (pH 7.3) without (A and C) and with 50 mM DHPC (B and D). The obtained spectra for apoE2-, apoE3-, and apoE4-(72-166) are shown as circles, triangles and squares, respectively. Solid lines show the best fit by CDSSTR (A and B) or the two-state unfolding model (C and D) (Eq. 1, see Materials and Methods). The parameters by best fit are shown in Table S1 and 1, respectively.


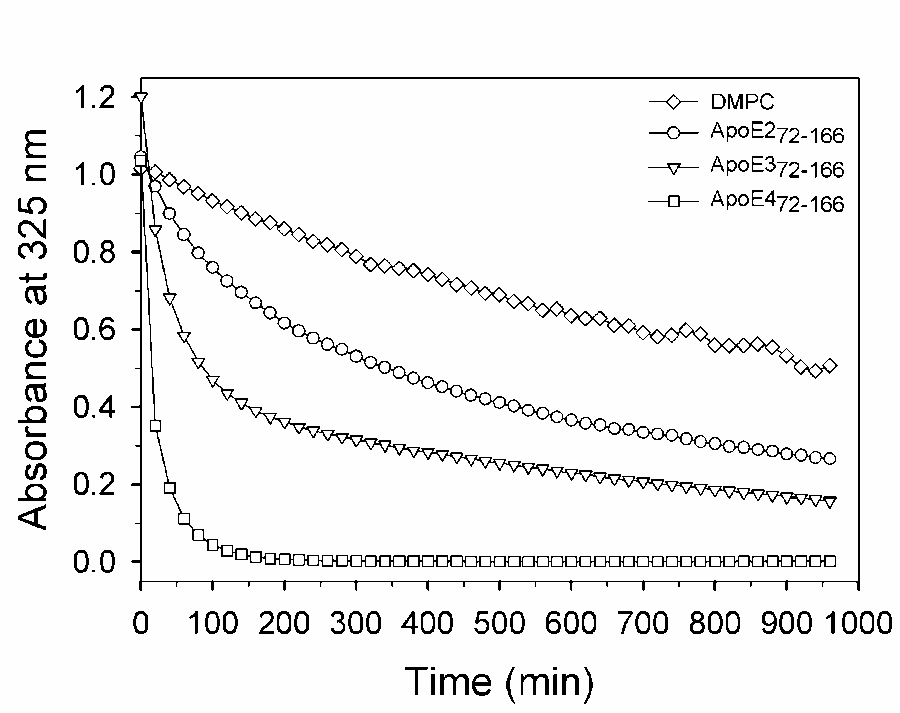


**Figure S2. DMPC turbidity clearance assay.** DMPC without apoE was shown as diamonds. Circles, DMPC with apoE2-(72-166); triangles, DMPC with apoE3-(72-166); squares, DMPC with apoE4-(72-166). The solid line indicated the fitting curve by biexponential decay equation (Eq. 4). Data of apoE3-(72-166) and apoE4-(72-166) are from [22]. The parameters by best fit are shown in Table S2.

**Figure S3. LDLR binding assay.** ^3^H-LDL binding to the LDL receptor was taken as 100% (bar 1). LDL receptor binding in the presence of competitors was shown as percentage of the control. Incubation contained the following competitors: a 20-fold excess of unlabeled LDL (bar 2); 0.5 mg of DMPC mLV (bar 3). Bar 4-9 showed apoE-(72-166) peptides only or apoE-(72-166)-DMPC as competitors. Data of apoE3 and apoE4 (Bar 5-6 and 8-9) are from [22].
